# Supplementary material for: Kekulé Counts, Clar Numbers, and ZZ Polynomials for All Isomers of (5,6)-Fullerenes C52–C70
Source: Molecules. 2024 Aug 24;29(17):4013. doi: 10.3390/molecules29174013 (PMC11396526; doi:10.3390/molecules29174013)
Supplement: Supplementary file 1 [file molecules-29-04013-s001.zip › Supplementary Materials/Figure S2.old.pdf]

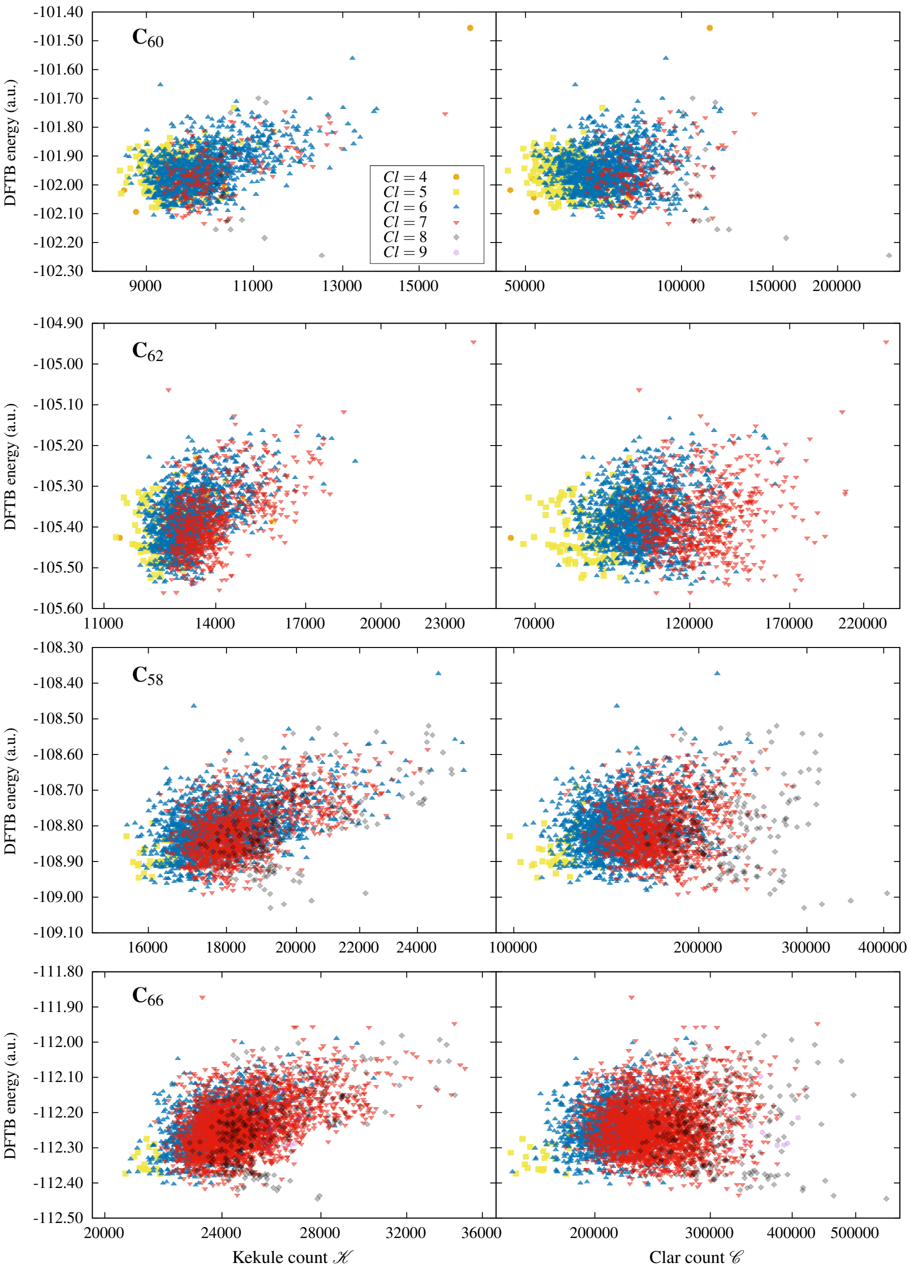

Figure S2: Total DFTB energies of all isomers of C_{60} and C_{66} plotted as a function of their topological invariants: Kekulé count $\mathcal{K}$ (left panels) and Clar count $\mathcal{C}$ (right panels). The C_{60}, C_{62}, C_{64}, and C_{66} systems are presented from top to bottom. The information about the Clar number Cl is conveyed via the symbol code explained in the legend. The plots show weak anti-correlation between E and $\mathcal{K}$ and no correlation betwen E and $\mathcal{C}$.
