# Supplementary figures and images for: Kekulé Counts, Clar Numbers, and ZZ Polynomials for All Isomers of (5,6)-Fullerenes C52–C70
Source: Molecules. 2024 Aug 24;29(17):4013. doi: 10.3390/molecules29174013 (PMC11396526; doi:10.3390/molecules29174013)

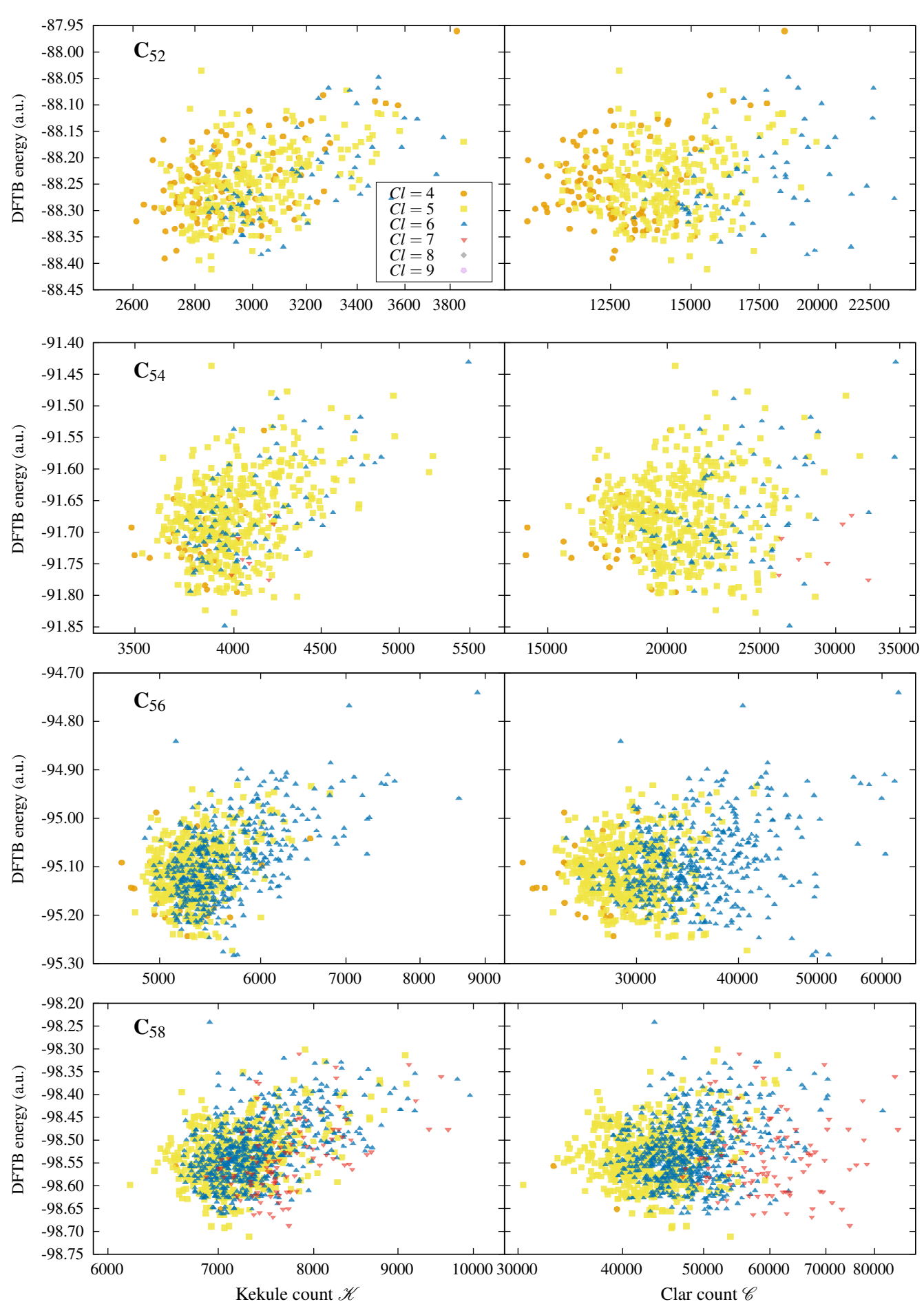

Supplement: Supplementary file 1 [file molecules-29-04013-s001.zip › Supplementary Materials/c52-58KClog.pdf]

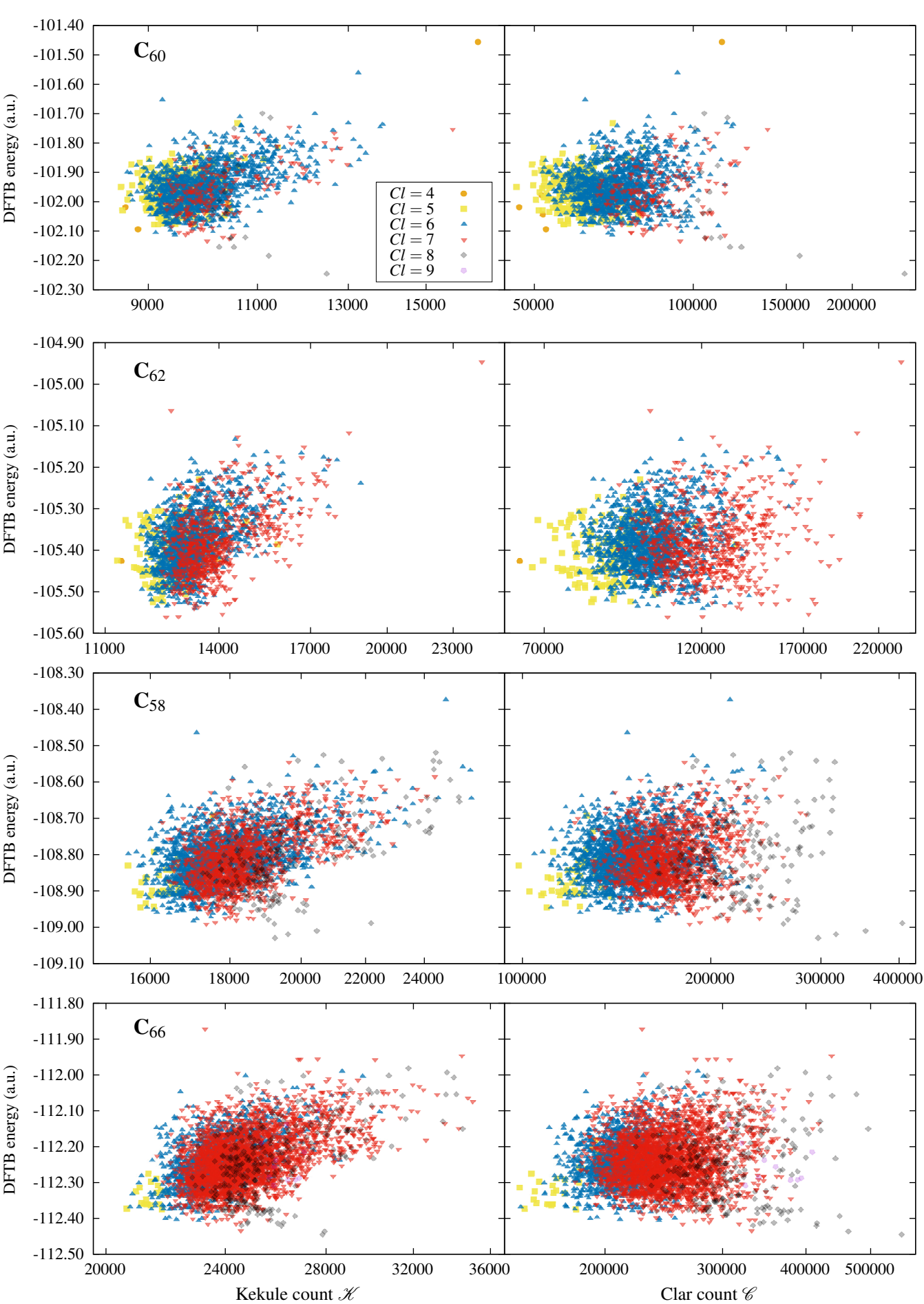

Supplement: Supplementary file 1 [file molecules-29-04013-s001.zip › Supplementary Materials/c60-66KClog.pdf]

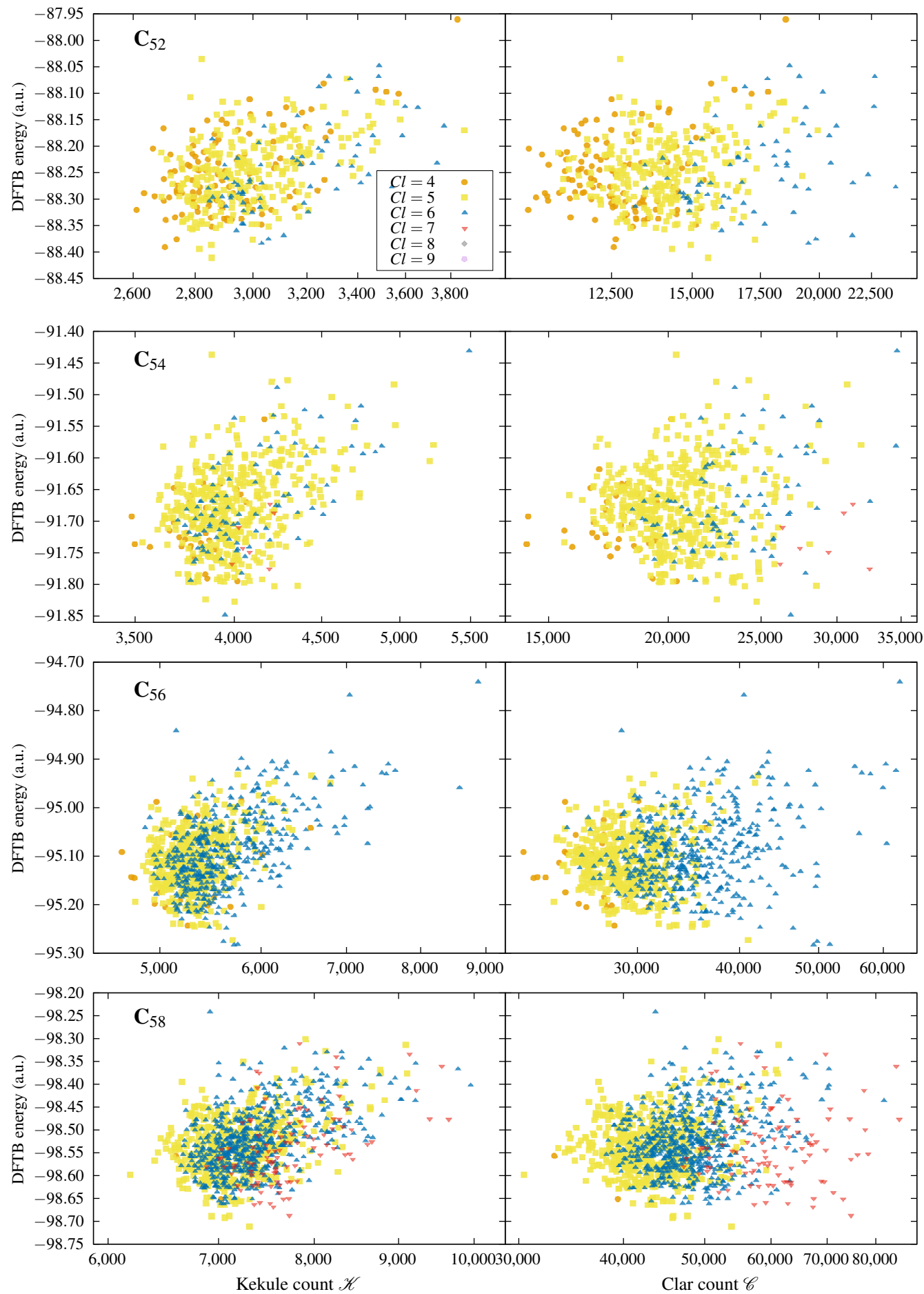

Supplement: Supplementary file 1 [file molecules-29-04013-s001.zip › Supplementary Materials/Figure S1.pdf]

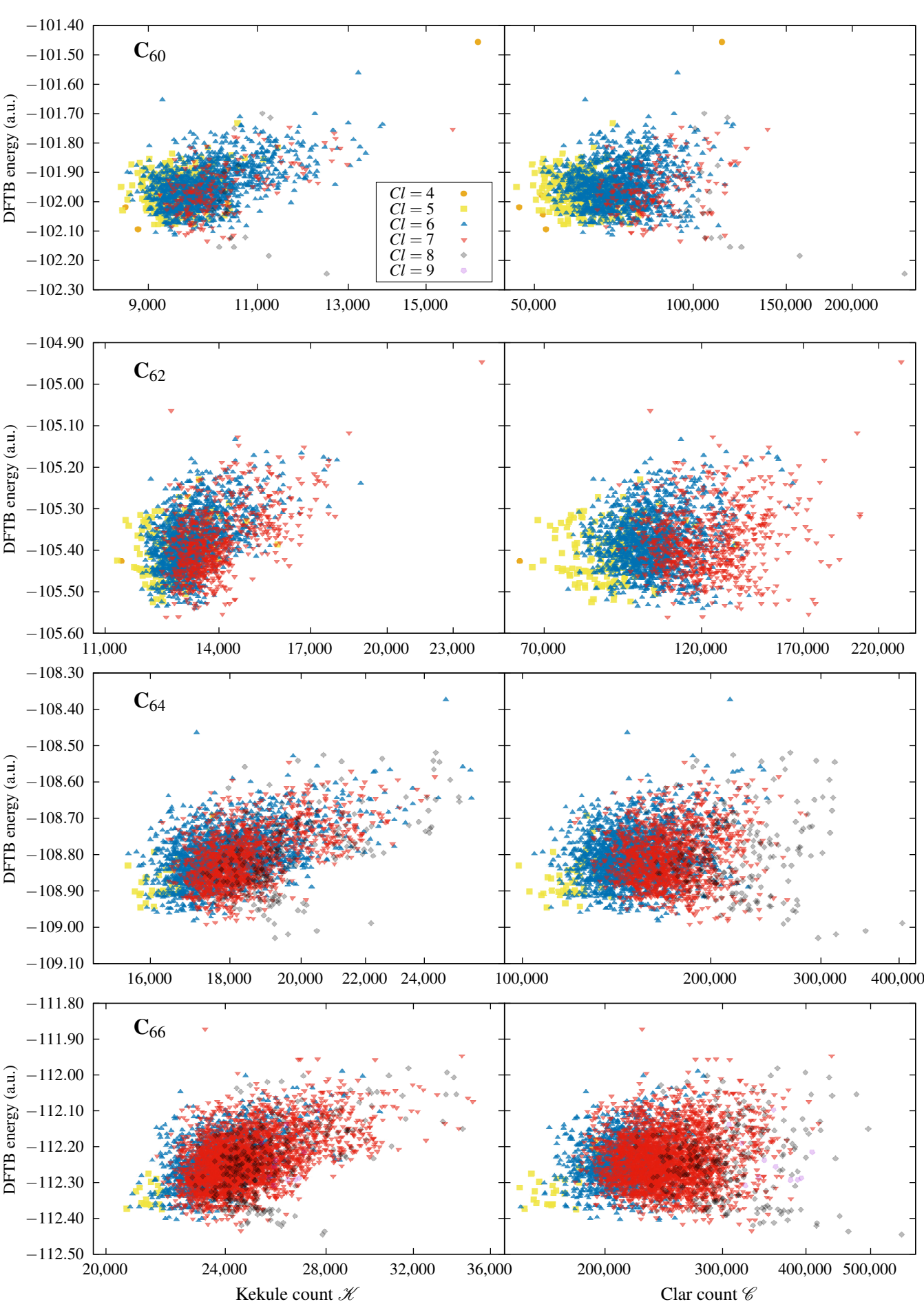

Supplement: Supplementary file 1 [file molecules-29-04013-s001.zip › Supplementary Materials/Figure S2.pdf]

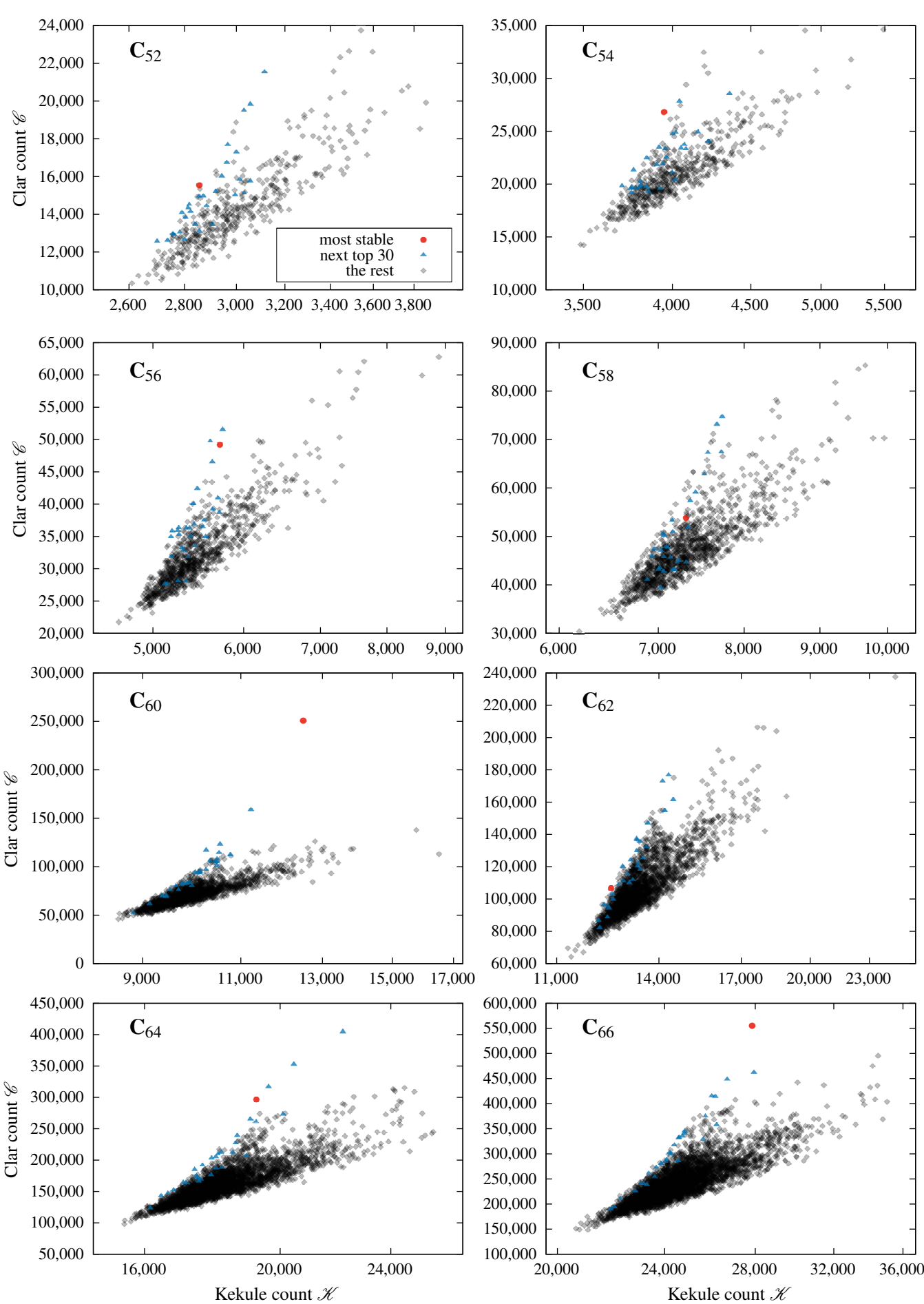

Supplement: Supplementary file 1 [file molecules-29-04013-s001.zip › Supplementary Materials/Figure S3.pdf]
